# Chronotype and Adherence to the Mediterranean Diet in Obesity: Results from the Opera Prevention Project

**DOI:** 10.3390/nu12051354

**Published:** 2020-05-09

**Authors:** Giovanna Muscogiuri, Luigi Barrea, Sara Aprano, Lydia Framondi, Rossana Di Matteo, Daniela Laudisio, Gabriella Pugliese, Silvia Savastano, Annamaria Colao, on behalf of the Opera Prevention Project

**Affiliations:** 1 Department of Clinical Medicine and Surgery, Endocrinology unit, Federico II University, Via Sergio Pansini 5, 80131 Naples, Italy; luigi.barrea@unina.it (L.B.); saraaprano@hotmail.com (S.A.); lydiaframondi@gmail.com (L.F.); rossanadimatteo96@hotmail.com (R.D.M.); dani.laudisio@libero.it (D.L.); robiniapugliese@gmail.com (G.P.); sisavast@unina.it (S.S.); colao@unina.it (A.C.); 2 Centro Italiano per la cura e il Benessere del paziente con Obesità (C.I.B.O), Federico II University, Via Sergio Pansini 5, 80131 Naples, Italy; 3 UNESCO Chair “Education for Health and Sustainable Development”, Federico II University, 80131 Naples, Italy

**Keywords:** Mediterranean diet, chronotype, obesity, lifestyle, sleep

## Abstract

Chronotype is the attitude of a subject in determining individual circadian preference in behavioral and biological rhythm relative to the external light–dark cycle. Obesity and unhealthy eating habits have been associated with evening chronotype. The Mediterranean diet (MD) is a healthy nutritional pattern that has been reported to be associated with better health and quality of sleep. Thus, the aim of the study was to investigate the association of chronotype categories with adherence to the MD in a population of middle-aged Italian adults. This cross-sectional study included 172 middle-aged adults (71.5% females; 51.8 ± 15.7 years) that were consecutively enrolled in a campaign to prevent obesity called the OPERA (obesity, programs of nutrition, education, research and assessment of the best treatment) Prevention Project that was held in Naples on 11–13 October 2019. Anthropometric parameters, adherence to the MD and chronotype were studied. Chronotype was classified as morning in 58.1% of subjects, evening in 12.8% and intermediate in 28.1%. Our results demonstrated that individuals with evening chronotype, when compared to intermediate (*p* < 0.001) and morning chronotype (*p* < 0.001), were more prone to follow unhealthy lifestyle, performing less regular activity and being more frequently smokers. In addition, they showed the lowest adherence to the MD compared to morning (*p* < 0.001) and intermediate chronotypes (*p* < 0.001). The lower the chronotype score, the higher body mass index (BMI) values in the whole population (r = −0.158; *p* = 0.038), thus suggesting that evening chronotype was a common finding in subjects with obesity. In addition, positive correlations of chronotype score with age (r = 0.159; *p* = 0.037) and PREDIMED score (r = 0.656; *p* < 0.001) were found. The adherence to the MD, more than the intake of the single food items, was found to predict morning and evening chronotypes. In conclusion, evening chronotype was associated with unhealthy lifestyle and low adherence to the MD. Chronotype score was inversely associated to BMI and positively associated to age and adherence to the MD. Thus, the assessment of chronotype should be taken into account in the management of obesity and in the development of nutritional strategies.

## 1. Introduction

Chronotype is the attitude of a subject determining individual circadian preference in behavioral and biological rhythm relative to the external light–dark cycle [1,2]. There are three general categories of chronotype that are based on the circadian behavioral phenotype variants: morning, evening and intermediate chronotypes [3]. Morning chronotype has a tendency to wake up early and prefers activities earlier in the day, while evening chronotype usually wakes up later and prefers to time peak activity during the late afternoon or evening. Intermediate chronotype is in an intermediate position between morning and evening chronotype. Evening chronotype has a tendency to have more health problems such as psychological disorders, gastrointestinal diseases and greater mortality compared to morning chronotype [4]. Furthermore, evening chronotype has been reported to be more prone to develop metabolic diseases such as type 2 diabetes and metabolic syndrome than morning chronotype [5]. Recent evidence from animal trials reported that eating at inappropriate circadian times may contribute to a poorer weight management and metabolic health [6,7,8]. Furthermore, in evening chronotype, sleep disturbances are a common finding because subjects with evening chronotype are used to sleep later in the night but need to wake up earlier than their biological morning due to social demands [9]. In most of the cases, sleep disturbances are associated with increased food intake, in particular unhealthy food, thus predisposing to the risk of obesity [10,11,12]. In a cross-sectional study performed in 52 female volunteers, it was found that late sleepers had a shorter sleep duration, later sleep onset and offset and meal times. Late sleepers have been found to consume more calories at dinner and after 8:00 PM, to have higher fast food, full-calorie soda and lower fruit and vegetable consumption. Consequently, higher body mass index (BMI) was associated with shorter sleep duration, later sleep timing, caloric consumption after 8:00 PM, and fast food meals [13]. In addition, individuals with evening chronotype have been found to consume more unhealthy foods, such as soft drinks and chocolate, and less healthy foods, such as vegetables, fruits and fish compared to those with morning chronotype [14,15]. In agreement with this, Mota et al. performed a cross-sectional study in 72 resident physicians assessing chronotype, food intake pattern, physical activity level, sleep quality and quantity and sleepiness. They found that the chronotype score was negatively associated with cholesterol, sweets and vegetables intakes. Furthermore, the chronotype score was positively associated with leisure-time index and physical activity [14]. This finding was also confirmed in pregnant women. Indeed, a cross-sectional study carried out in 100 pregnant women in the first gestational trimester (≤12 weeks of gestation) reported that morning chronotype was associated with a better quality of diet [15]. Another characteristic of evening chronotype is a shift of food consumption toward later times in the day and higher evening energy intake [16]. In addition, a cross-sectional Japanese study reported that evening chronotype was also associated with low vitamin and mineral intakes. This study was performed in 112 Japanese young women showing a significant association of evening chronotype with a lower energy-adjusted intake of protein, calcium, magnesium, zinc, vitamins (D, riboflavin, and B6), and vegetables, and with a higher intake of noodles [17]. Given the negative effect of evening chronotype on nutrition, we aimed to investigate the potential association of chronotype categories with adherence to the Mediterranean diet (MD) and the consequent effects on anthropometric parameters in a population of Italian middle-aged adults, who are culturally attached to this dietary pattern. 

## 2. Materials and Methods 

### 2.1. Study Design, Settings, Participants and Protocol

One-hundred seventy-two middle-aged subjects aged 51.8 ± 15.6 years were recruited in a cross-sectional study during the OPERA (obesity, programs of nutrition, education, research and assessment of the best treatment) Prevention Project held in Naples on 11–13 October 2019 (mettere ref). [18] The OPERA Prevention Project is part of the great event Campus 3S (health, sport and solidarity), an outdoor hospital which has moved since 2010 from Naples to the largest Italian squares. (www.campussalute.it for details). Campus 3S aims to screen the health status of the general population through free consultations, visits, and diagnostics for people coming to the outdoor hospital held in different public squares of Italy. The OPERA Prevention Project is also a strategic project of the UNESCO Chair on “Health Education and Sustainable Development” (https://www.unescochairnapoli.it/ for details). The OPERA Prevention Project provided an innovative free path by which subjects with obesity were visited and received the right advices to start a weight loss program. All subjects gave their written informed consent to the study that was carried out in agreement with the Helsinki declaration for human studies. Recruitment consisted of an informative talk, explaining details to the subjects about the research, and encouraging them to take part to the study. Eligible participants for the study were middle-aged subjects having normal liver, cardiopulmonary and kidney functions as determined by self-reported medical history. In addition, we excluded subjects taking medications for hepatic, renal and cardiopulmonary diseases. Other exclusion criteria were alcohol abuse assessed as previously reported [19] and/or drug abuse assessed performing a thorough evaluation in order to detect an inability to control the use of drugs and history of allergy or intolerance to any food components of the MD. Subjects following a specific dietary regimen for any reason were also excluded from the study. 

### 2.2. Sample Size Justification and Power

The sample size was determined using the software ClinCalc tool (www.clincalc.com) based on the results from Yu et al. [5], considering the prevalence of morning and evening chronotypes as the main variables. A statistical power (1-β) of 95% and a level of significance (α) of 5% were considered which resulted in a sample of 130 subjects as the necessary number of subjects to be enrolled for this study. However, we considered to enroll 10% more subjects in order to compensate for any drop-out. During the campaign, 172 were considered eligible for the study and since this sample not only satisfied the minimum necessary number to be enrolled but also could provide further strength to the results, we decided to include all of them in the statistical analysis.

### 2.3. Data Collection

Trained nutritionists assessed anthropometric parameters, asked questions regarding demographic information and lifestyle habits. We defined “current smokers” when the subjects reported to smoke at least one cigarette per day and “non current smokers” when the subjects reported to not smoke. The participants habitually engaged in at least 30 min/day of aerobic exercise (YES/NO) were defined as physically active, as we have already fully reported in previous studies [20].

#### 2.3.1. Anthropometric Parameters

Subjects dressed in light clothes only and took their shoes off when anthropometric parameters were assessed, as already reported [21]. The formula of BMI was the following: weight (kg)/height (m^2^). A wall-mounted stadiometer was used to assess height. A calibrated scale was used to assess body weight. Waist circumference (WC) was measured to the closest 0.1 cm with a nonextensible tape. Grade I Obesity was defined if BMI ranges from 30 to 34.9 kg/m^2^, grade II obesity if BMI ranges from 35 to 39.9 kg/m^2^ and grade III obesity if BMI was equal to or greater than 40.0 kg/m^2^.

#### 2.3.2. Adherence to the Mediterranean Diet

As already reported [22], the adherence to the MD was evaluated using the previously validated 14-item PREDIMED (Prevención con Dieta Mediterránea) questionnaire [23]. A qualified nutritionist administered the questionnaire during a face-to-face interview to all the enrolled subjects. Briefly, for each item, scores 1 and 0 were assigned; PREDIMED score was calculated as follows: 0–5, lowest adherence; score 6–9, average adherence; and score ≥10, highest adherence [23].

#### 2.3.3. Assessment of Chronotype

Subject morningness–eveningness was measured with the Horne–Ostberg Morningness–Eveningness Questionnaire (MEQ) [5]. The MEQ consisted of 19 questions about preferred sleep time and daily performance. The scores ranged from 16 to 86. Based on their scores, individuals were categorized as being a morning (59–86), neither (42–58), or evening (16–41) chronotype. 

### 2.4. Statistical Analysis

The data distribution was evaluated by Kolmogorov–Smirnov test and data not normally distributed were normalized by logarithm. The chi square (χ^2^) test was used to determine the significance of differences in frequency distribution of smoking habit, physical activity, BMI categories, adherence to the MD and dietary components included in the PREDIMED questionnaire. Differences among chronotypes were analyzed by a between-group analysis of variance (ANOVA) test followed by the Bonferroni post hoc test. The correlations between study variables were performed using Pearson r correlation coefficients. In addition, a multiple linear regression analysis model (stepwise method), expressed as R^2^, Beta (β) and t, with chronotype score as dependent variable, was used to estimate the predictive value of food items (use of extra virgin olive oil as main culinary lipid, extra virgin olive oil >4 tablespoons, vegetables ≥2 servings/day, fruits ≥3 servings/day, red/processed meats <1/day, butter, cream, margarine <1/day, soda drinks <1/day, wine glasses ≥7/week, fish/seafood ≥3/week, commercial sweets and confectionery ≤2/week, tree nuts ≥3/week, poultry more than red meats) and PREDIMED score. Data were collected and analyzed using the MedCalc^®^ package (Version 12.3.0 1993-2012-Mariakerke, Belgium). 

## 3. Results

The study population consisted of 172 participants (71.5% females; 51.8 ± 15.7 years). In Table 1 sociodemographic, anthropometric characteristics, adherence to the MD and chronotypes were reported. Obesity was present in most of the enrolled subjects. In particular, grade I obesity was detected in 58 subjects (33.7%), grade II obesity in 29 subjects (16.9%) while grade III obesity was detected in 20 individuals (11.6%). 146 (84.8%) had smoking habits and 88 (51.2%) performed moderate physical activity. The mean PREDIMED score was 7.8 ± 2.2. Twenty-one (12.2%) subjects had low adherence, 110 (64%) had average adherence while 41 (23.8%) had high adherence to the MD. The mean score of chronotype was 58.4 ± 13.1. One-hundred (58.1%) subjects had morning chronotype, 50 (29.1%) subjects had intermediate chronotype while 22 (12.8%) evening chronotype.

Participants with intermediate chronotype were younger than those with morning and evening chronotype. Evening chronotype were less prone to follow healthy lifestyle; indeed, they did less regular exercise and most of them were smokers. Evening chronotype had the lowest PREDIMED score, i.e., they had the lowest adherence to the MD compared to the other chronotypes. Evening chronotype had the highest percentage of subjects with low adherence to the MD and the lowest percentages of subjects with average and high adherence to the MD, as shown in Table 2.

### Correlation Studies

Correlation analyses were performed to assess the association of chronotype score with age and anthropometric parameters. Chronotype score was positively associated to age (r = 0.159, *p* = 0.04) and inversely correlated to BMI (r = −0.16, *p* = 0.04). No correlation was found between WC and chronotype (r = −0.04, *p* = 0.57). Table 3 showed correlations among chronotype score with age and anthropometric parameters after adjustment for gender and smoking.

**Figure 1:**
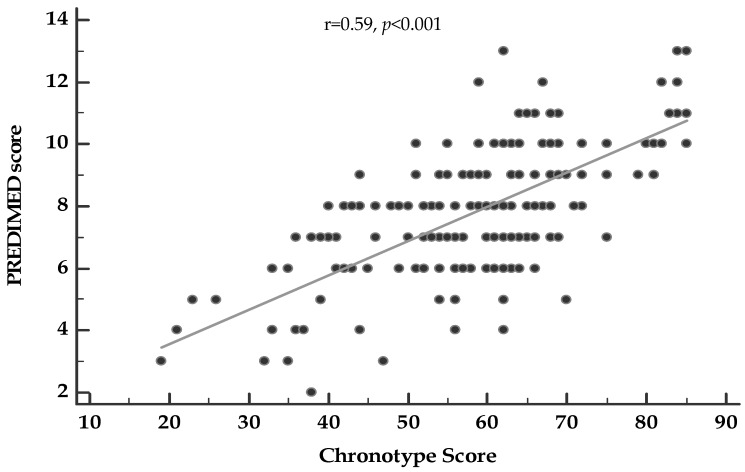
Correlation between chronotype score and PREDIMED score A *p*-value denotes a significant difference (*p* < 0.05).

Figure 1 showed the correlation between chronotype score and PREDIMED score, adjusted for gender and smoking. Chronotype score was positively associated to PREDIMED score also after adjustment for covariates. 

Table 4 shows the results of the bivariate proportional odds ratio model performed to assess the association of chronotype score with food items of the PREDIMED questionnaire. Extra-virgin olive oil (EVOO), vegetable, fruit, fish, poultry, nuts and wine consumption were positively associated to chronotype score while the highest odds of soda drinks, red meats, butter, cream, margarine commercial sweets and confectionery seemed to have a negative effect. 

In Table 5 a bivariate proportional odds ratio model was performed to assess the association of chronotype categories with the adherence to the MD. As expected, the adherence to the MD was associated with morning, neither and evening chronotype.

To assess the most predictive factor of chronotype score among the single PREDIMED items (use of extra virgin olive oil (EVOO) as main culinary lipid, extra virgin olive oil >4 tablespoons, vegetables ≥2 servings/day, fruits ≥3 servings/day, red/processed meats <1/day, butter, cream, margarine <1/day, soda drinks <1/day, wine glasses ≥7/week, fish/seafood ≥3/week, commercial sweets and confectionery ≤2/week, tree nuts ≥3/week, poultry more than red meats) and PREDIMED score, we performed a multiple linear regression analysis model, including these parameters. PREDIMED score entered at the first step appeared to exert a powerful influence on morning (*p* < 0.001) and evening chronotype scores (*p* = 0.02) while butter, cream, and margarine <1/day influenced intermediate chronotype (*p* = 0.04), as shown in Table 6. 

## 4. Discussion

Evening chronotype was present in 12.8% of the middle-aged general population and was associated with the lowest adherence to the MD compared to intermediate and morning chronotype. The lower the chronotype score, the higher the BMI in the whole population. In addition, the chronotype score positively correlated to age and to PREDIMED score. The main determinant of morning and evening chronotype categories was the cluster of food enclosed in the MD, instead of the single food items. The main determinant of intermediate chronotype was butter, cream, margarine <1/day. As well known, obesity is a growing health problem. Worldwide research studies tried to find the best nutritional approaches to tackle obesity. Several studies reported that individuals with evening chronotype have a less healthy lifestyle than those with morning chronotype [24,25]. This finding was confirmed in our population in which those with evening chronotype were less prone to perform regular physical activity, and most of them were smokers. In addition, we found that the lower the chronotype score, the higher the BMI, thus suggesting that evening chronotype (the chronotype’s category with the lowest range score) was more frequently associated with obesity [26,27]. Soreca et al. performed a cross-sectional study in 29 patients with bipolar I disorder not currently experiencing an affective episode, and found that patients with evening chronotype have a higher percentage of total body fat [26]. Similar findings were detected in a cross-sectional study carried out by Ruiz-Lozano et al. in 252 subjects with severe obesity undergoing bariatric surgery. Subjects with evening chronotype showed significantly higher initial body weight and BMI than those with morning chronotype. Furthermore, individuals with evening chronotype, when compared to those with morning chronotype, lost less weight (percentage of excess weight loss) after bariatric surgery. Weight-loss progression between the two chronotype groups was significantly different from the fourth year after the bariatric surgery, with a higher weight regain among patients with evening type [27]. Data coming from the Sleep Extension Study suggested that evening chronotype linked to higher BMI and neck circumference in a population that included both obese and severely obese subjects; in addition, subjects with evening chronotype ate later and had a tendency towards fewer and larger meals [28]. One of the hypotheses that could explain the association between evening chronotype and obesity could lie in the fact that those with evening chronotype are more prone to follow an unhealthy diet. Indeed, we found that subjects with evening chronotype had lower adherence to the MD compared to the two other categories. Our findings were in agreement with a previous cross-sectional study performed in 416 middle-aged adults aiming at investigating the contribution of chronotype to abdominal fat distribution and collaterally reporting a higher adherence to the MD in subjects with morning chronotype compared to those with intermediate and/or evening chronotype [29]. However, in this study the authors investigated the association between the adherence to the MD in terms of PREDIMED score and chronotype categories. In our study, we further investigated if there was a single food or a cluster of the food enclosed in the MD whose intake could be associated to chronotype categories. Interestingly, we found that the most powerful predictive factor of morning and evening chronotypes was the cluster of foods belonging to the MD instead of a single food. Of course, morning chronotype had a higher adherence to the MD, and conversely, evening chronotype a lower adherence, thus suggesting that morning or evening chronotype could determine an attitude in following a nutritional pattern in its entirety that is healthy in the case of morning chronotype and that is unhealthy in the case of evening chronotype. Another hypothesis could be that the intake of the whole cluster of the MD could have a role in determining the chronotype more than single food intake. Indeed, polyunsaturated fatty acids (PUFA) and polyphenols have been described to have several neuroprotective properties such as neuroprotection via modulation of neural mediators and modulation of different signaling pathways that could contribute to a better mental health (i.e., depressive symptoms, cognitive impairment, etc.) [30,31,32]. Furthermore, a high adherence to the MD could also contribute to the restoration of a normal sleeping pattern, that in turn plays an important role in determining the chronotype [3]. We found that intermediate chronotype was associated with butter, cream, margarine <1/day, thus suggesting that saturated fats could have a role in determining this chronotype. The strengths of this study include a random sample and a population-based approach. Furthermore, chronotype was assessed using a validated method (3) and analyses were adjusted for several potential confounding factors. However, this study also has certain limitations. The cross-sectional design can not reveal causality. Furthermore, dietary intakes and the assessment of chronotype were based only on self-reported data. Thus, possible memory and reporting biases may affect the data to some extent. In addition, people with obesity in particular often misreport their food intake, and furthermore, health-conscious people are more likely to participate in health surveys. The study population was unbalanced in regard to gender, smoker status and age group representing only middle-aged people, although we minimized this bias by adjusting the statistical analysis for these potential confounding factors.

## 5. Conclusions

In conclusion, we reported that evening chronotype was associated with the lowest adherence to the MD compared to intermediate and evening chronotype. The lower the chronotype score, the higher the BMI in the whole population; in addition, the main determinant of morning and evening chronotype scores was the cluster of the food enclosed in the MD, instead of single food items. Further research should explore whether investigating chronotype within the context of adherence to the MD potentially being part of an overall healthier lifestyle pattern; this topic should be investigated with a prospective and longitudinal study design. Finally, experimental studies are needed to investigate the impact of chronotype on health and dietary intake, allowing for the investigation on causality and mechanisms. 

## Figures and Tables

**Table 1:** Lifestyle habits, anthropometric measurements, adherence to the Mediterranean diet (MD) and chronotype categories and score.

Parameters	Study Population *n* = 172
Age	51.8 ± 15.7 years
**Gender**	
Males	49, 28.5%
Females	123, 71.5%
**Lifestyle Habits**	
**Smoking**	
Yes (*n*, %)	146, 84.8%
No (*n*, %)	26, 15.2%
Physical activity	
Sedentary (*n*, %)	84, 48.8%
Moderate (*n*, %)	88, 51.2%
Anthropometric measurements	
Weight (kg)	84.6 ± 18.9
Height (m)	1.6 ± 0.09
BMI (kg/m^2^)	32.1 ± 6.3
Normal-weight (*n*, %)	18, 10.5%
Over-weight (*n*, %)	47, 27.3%
Obesity I (*n*, %)	58, 33.7%
Obesity II (*n*, %)	29, 16.9%
Obesity III (*n*, %)	20, 11.6%
WC (cm)	103.0 ± 16.0
Adherence to the MD	
PREDIMED score	7.8 ± 2.2
Low adherence to the MD	21, 12.2%
Average adherence to the MD	110, 64.0%
High adherence to the MD	41, 23.8%
Chronotype Score	58.4 ± 13.1
Morning Chronotype	100, 58.1%
Intermediate Chronotype	50,29.1%
Evening Chronotype	22,12.8%

WC: waist circumference; MD: Mediterranean diet; BMI: body mass index; PREDIMED: adherence to the Mediterranean diet (Prevención con Dieta Mediterránea).

**Table 2:** Lifestyle habits, anthropometric measurements, adherence to the Mediterranean diet (MD) according to chronotype categories.

Parameters	Morning Type *n* = 100, 58.1%	Neither Type *n* = 50, 29.1%	Evening Type *n* = 22, 12.8%	*p*-Value
**Gender**				
Males	34, 34.0	13, 26.0	2, 9.1	*p* = 0.18
Females	66, 66.0	37, 74.0	20, 90.9	
**Lifestyle Habits**				
Age (years)	55.5 ± 13.7	43.0 ± 17.4 ^a,b^	55.3 ± 11.9	<0.001
**Smoking**				
Yes (*n*, %)	7, 7.0	11, 22.0	8, 36.4 ^a,c^	*p* < 0.001
No (*n*, %)	93, 93.0	39, 78.0	14, 63.6 ^a,c^	
**Physical activity**				
Sedentary (*n*, %)	39, 39.0	26, 52.0	19, 86.4 ^a,c^	*p* < 0.001
Moderate (*n*, %)	61, 61.0	24, 48.0	3, 13.6 ^a,c^	
**Anthropometric measurements**				
Weight (kg)	82.9 ± 19.0	88.1 ± 20.6	83.7 ± 12.5	0.29
Height (m)	1.6 ± 0.09	1.6 ± 0.08	1.6 ± 0.08	0.57
BMI (kg/m^2^)	31.4 ± 5.8	33.1 ± 7.3	32.6 ± 5.5	0.27
Normal-weight (*n*, %)	10, 10.0	7, 14.0	1, 4.5	*p* = 0.47
Over-weight (*n*, %)	33, 33.0	9, 18.0	5, 22.7	*p* = 0.13
Obesity I (*n*, %)	32, 32.0	15, 30.0	11, 50.0	*p* = 0.22
Obesity II (*n*, %)	15, 15.0	11, 22.0	3, 13.6	*p* = 0.51
Obesity III (*n*, %)	10, 10.0	8, 16.0	2, 9.1	*p* = 0.52
WC (cm)	102.7 ± 16.4	102.9 ± 17.3	104.5 ± 11.8	0.89
**Adherence to the MD**				
PREDIMED score	8.8 ± 1.9	7.0 ± 1.5 ^a,b^	5.1 ± 1.8 ^a,c^	<0.001
Low adherence to the MD	3, 3.0	6, 12.0 ^a,b^	12, 54.5 ^a,c^	*p* < 0.001
Average adherence to the MD	58, 58.0	42, 84.0 ^a,b^	10, 45.5 ^a,c^	*p* = 0.001
High adherence to the MD	39, 39.0	2, 4.0 ^a,b^	0, 0 ^a,c^	*p* < 0.001

^a^*p* < 0.05 vs. morning type; ^b^ *p* < 0.05 vs. evening type; ^c^ *p* < 0.05 vs. intermediate type; WC: waist circumference; MD: Mediterranean diet; BMI: body mass index; PREDIMED: adherence to the Mediterranean diet (Prevención con Dieta Mediterránea).

**Table 3:** Correlations of chronotype score with age and anthropometric parameters after adjustment for gender and smoking.

	Chronotype Score	
Parameters	r	*p*-Value
Age (years)	0.21	0.01
BMI (kg/m^2^)	−0.18	0.02
WC (cm)	−0.07	0.35

WC: waist circumference; BMI: body mass index.

**Table 4:** Bivariate proportional odds ratio models performed to assess the association of chronotype score with the dietary components included in the PREDIMED questionnaire and with PREDIMED categories.

Questions	OR	R^2^	95% IC	*p*-Value
Use of extra virgin olive oil (EVOO) as main culinary lipid	1.07	0.07	1.03–1.12	0.001
Extra virgin olive oil >4 tablespoons	1.03	0.04	1.00–1.06	0.013
Vegetables ≥2 servings/day	1.05	0.07	1.02–1.07	0.001
Fruits ≥3 servings/day	1.07	0.13	1.04–1.10	<0.001
Red/processed meats <1/day	1.05	0.08	1.02–1.08	<0.001
Butter, cream, margarine <1/day	1.05	0.06	1.02–1.08	0.001
Soda drinks <1/day	1.04	0.07	1.01–1.07	0.001
Wine glasses ≥7/week	1.05	0.05	1.01–1.09	0.004
Legumes ≥3/week	1.02	0.02	0.99–1.05	0.08
Fish/seafood ≥3/week	1.03	0.03	1.00–1.06	0.02
Commercial sweets and confectionery ≤2/week	1.04	0.04	1.01–1.06	0.007
Tree nuts ≥3/week	1.03	0.03	1.00–1.06	0.014
Poultry more than red meats	1.05	0.08	1.03–1.08	<0.001
Use of sofrito sauce ≥2/week	1.02	0.01	0.99–1.04	0.22
**PREDIMED categories**				
Low adherence to the MD	0.89	0.20	0.85–0.93	<0.001
Average adherence to the MD	0.99	0.01	0.96–1.01	0.05
High adherence to the MD	1.15	0.25	1.09–1.21	<0.001

**Table 5:** Bivariate proportional odds ratio models performed to assess the association of PREDIMED score with the chronotype categories.

Chronotype Categories	OR	R^2^	95% IC	*p*-Value
Morning Type	2.02	0.29	1.61–2.54	<0.001
Neither Type	0.78	0.06	0.66–0.91	0.002
Evening Type	0.42	0.22	0.30–0.59	<0.001

**Table 6:** Multiple regression analysis models (stepwise method) with chronotype score as a dependent variable to estimate the predictive value of the single PREDIMED items and PREDIMED score.

Parameters	Multiple Regression Analysis			
**Model 1: Morning Type**	**R^2^**	***β***	**t**	***p*-Value**
*PREDIMED score*	0.18	0.43	4.69	<0.001
**Model 2: Neither Type**				
*Butter, cream, margarine <1/day*	0.09	−0.30	−2.17	0.04
**Model 3: Evening Type**				
*PREDIMED score*	0.23	0.48	2.46	0.02

## Abbreviations

MD: Mediterranean diet; BMI: body mass index; OPERA: Obesity, Programs of Nutrition, Education, Research and Assessment of the beast treatment; WC: Waist circumference; PREDIMED: Prevención con Dieta Mediterránea; MEQ: Morningness-Eveningness Questionnaire; EVOO: Extra-virgin olive oil; PUFA: Polyunsaturated fatty acids.
